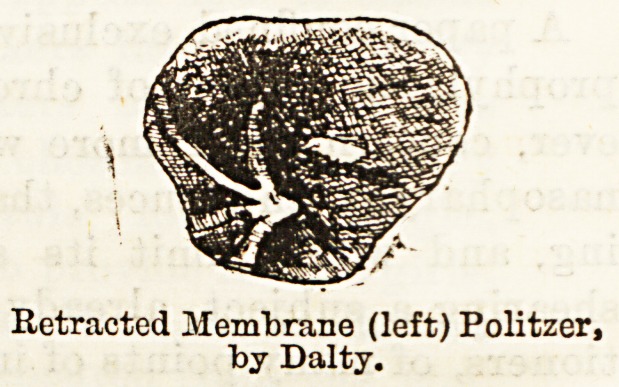# Chronic Catarrh of the Middle Ear

**Published:** 1894-07-28

**Authors:** L. H. Pegler


### Medical Progress and Hospital Clinics.

[*The Editor will be glad to receive offers of co-operation and contributions from members of the profession. All letters should be addressed to* The Editor, The Lodge, Porchester Square, London, W.]

### CHRONIC CATARRH OF THE MIDDLE EAR.

By L. H. Pegler, M.D., M.R.C.S.

Introductory.

**Importance of Early Recognition.**—The purpose of this paper is to direct attention to the great prevalence of chronic imperforate (popularly known as throat) deafness amongst the youthful and adult members of our population. There may be considerable diversity of opinion as to the advancement that has been made in recent years in the cure of this rebellious malady when once established; there can, however, be little dispute as to the encouraging results that wait upon the treatment of conditions affecting those regions and organs that border upon the ear, and stand to perversion of function in the latter in the relation of cause and effect. So that the unsatisfactory character of our efforts to modify organic changes in the tympanic cavity must render it evident to all, how emphatic should be our endeavours to anticipate such changes by dealing early and effectually with the precursors of deafness in parts that are within easy access, and respond favourably to surgical procedures.

A paper confined exclusively to the etiological and prophylactic aspect of chronic deafness would, however, concern itself more with diseases of the nose, nasopharynx and fauces, than with the organ of hearing, and would limit its scope too much, besides shearing a subject, already dry enough to most practioners, of many points of interest. We shall attempt therefore in preference to engage the attention of those of our readers who have not made this a special study by describing somewhat discursively in the first place the principal manifestations of inflammation of the middle ear, in order to better understand the position occupied in the series, by the particular form of it about to be considered. We shall next point out the chief foci of origin of the catarrhal process, so far as at present recognised, and conclude with a few remarks on diagnosis and treatment.

**Nomenclature of the Disease.**—The clinical features and treatment of chronic aural suppuration have already been discussed by the writer in a series of articles that appeared in this journal during October, 1893. It is partly on this account that we take occasion in the present instance to study the malady which afflicts nearly the whole remaining contingent of aural patients attending the hospital clinics, and from which, in fact, by far the larger proportion of the patients who are seen at the special throat hospitals suffer. Unlike the subjects of otorrhœa and perforation, who complain chiefly of a discharge from the ear, and as a rule say little about hardness of hearing unless specially interrogated, the cases about to be considered are those of patients who ask our advice and assistance for deafness principally, and oftener than not for noises in the head or giddiness also. It is not very easy to give a strictly accurate and comprehensive title to this affection, because the symptoms just mentioned may arise during some period in the course of more than one form of aural trouble; which though having a common catarrhal origin, and agreeing in preserving an inperforate drum membrane, differ a good deal in other important particulars. Owing to this fact, and to the existence of mixed and intermediate forms, not only the title, but also the classification of the disease and its varieties is a matter of considerable difficulty; and we accordingly find some disagreement as to the terms and methods of arrangement employed by the principal authors who describe it. We select the designation chronic catarrh of the middle ear as meeting the case fairly satisfactorily, and as offering an unmistakable distinction from that of suppurative inflammation of the middle ear already described.

Perhaps the distinction existing between the two diseases is not intrinsically so great as seems apparent at first sight, since it is really more a clinical than a pathological one; but we shall better appreciate the relationship between the acute and chronic forms of otitis media if we approach the consideration of the subject by passing briefly in review that of aural inflammation in general, and the divergencies pursued by the disease in the course of its various developments.

**Position of Chronic Catarrh in the Category of Aural Inflammations.**—We shall start with the assumption that catarrhal inflammation of the middle ear, as regards the pathological processes therein included, is identical in nature and causation with those of catarrh in the neighbouring respiratory tract, of which, as Sexton, who especially dwells upon this relationship, aptly expresses it, the Eustachian tube, tympanum, and mastoid cells, are but an outlying district, so that when any portion of this tract, extending from the nose to the larynx, is in a catarrhal state, there is a strong liability for the outlying portion to be attacked simultaneously or subsequently, just, in fact, as a rhinitis may be propagated from the nasal cavities to the maxillary, ethmoidal, sphenoidal, or frontal sinus; or a pharyngitis to the trachea and bronchial tubes. Or, again, this same district may be attacked primarily and independently.

There appears, however, to be a greater power of resistance to catarrhal invasion by the tissues of one district than by those of a neighbouring one, in certain individuals, and this accounts for the immunity enjoyed by the middle ear in the numerous instances we meet with, in which a pharyngitis is manifestly present and no aural complication follows, and *vice versa*.

By whatsoever means the middle ear has become the seat of attack, that is to say, whether primarily, as a result of one of the many exciting causes of catarrh acting upon the organism from without, or secondarily, by extension from the throat, the course followed by the inflammatory phenomena is liable to much variation. For example, there may be an effusion of fluid into the tympanic cavity, or this circumstance may be entirely absent. Supposing, again, an exudate exist, it may be mucous, tenacious or viscid, serous, purulent, or any combination of these conditions. An emigration of leucocytes taking place from the blood vessels of the affected parts, as a result of infection by pyogenic organisms, a secretion otherwise serous becomes purulent in character, just as a simple pleuritic effusion or an empyema may result upon an inflammation of the pleura.

This suppurative process (except in tuberculous patients, in whom its onset is insidious and painless) is generally ushered in by more or less constitutional disturbance and suffering. Moreover, pus formation to any considerable extent commonly implies extension of inflammation to the membrana tympani (myringitis), and if the vis a tergo is great and resolution or drainage by the Eustachian tube do not relieve the tension, perforation results. Hence, purulence occurring so frequently in conjunction with rupture of the drum-head, although it cannot be diagnosed clinically till perforation has taken place, serves as a convenient basis of classification, enabling us to distinguish roughly two divisions of otitis media, one which, for convenience, we may term the "inflammatory," in which there is purulence and acuteness of symptoms, with or without perforation, and a second or "catarrhal," in which the secretion contains but little if any pus, lesion of the membrane is rare, and the course is chronic and unmarked by constitutional disturbance.

Since the amount of secretion in this non-febrile group shades off imperceptibly to *nil*, it is useful to include under the same heading all the chronic catarrhs, including that sub-section in which secretion is absent altogether, for this is, strictly speaking, only a classification of types, the number of transition cases that occur, rendering absolute precision of arrangement impossible. The method adopted here is practically that of Van Tröltsch, and is taken exception to, amongst others especially by Roosa, who objects strongly to the term "'catarrh' being applied to a sunken drumhead, immovable chain of bones, dry pharynx, and easily permeable Eustachian tubes." He therefore designates the secreting form "catarrhal," and the dry "proliferous" inflammation.

**Acute Otitis Media.**—We need make no further allusion to the inflammatory group, which has been discussed elsewhere, beyond drawing attention to the non-perforating examples of the acute form (otitis media acuta of Politzer), which forms a connecting link with the catarrhs, and may assume many of the characters of the latter. As a general rule they run a milder course than the acute suppurative cases, and the drum membrane remains intact; but a termination such as the following is sometimes observed: The membrane, after showing evidence of intense congestion, assumes a greyish, leaden hue, and displays a peculiar wrinkling of its superficial epidermic layer. Pain and constitutional disturbance in the meantime may be very considerable. Perforation then takes place, and a thin serous or sero-purulent discharge escapes for a day or two, healing generally following rapidly. In other less favourable cases, again, the foundation is laid of chronic imperforate deafness. The "earaches" commonly complained of by children are examples of this form of otitis.

With this brief mention, therefore, of acute inflammation of the middle ear, the treatment of which is similar to that already directed for the early stages of acute suppuration, we turn now to a fuller consideration of the non-perforative catarrhs constituting our second division, in which pain and pyrexia are absent, and chronicity is an essential feature.

**The Secreting Forms of Middle Ear Catarrh.**—These comprise (1) recent mucous catarrh, or acute Eustachian obstruction, such as one often sees associated with a common cold, and including a more chronic form of the same occurring as an accompaniment of more permanent lesions in the respiratory passages; (2) adhesive catarrh, appearing either as a complication, or later development of the foregoing, or on the other hand, as an idiopathic inflammatory process, without effusion; the so-called dry catarrh of the middle ear (sclerosis).

A familiar and frequent example of simple mucous catarrh, which we may consider relatively speaking as the sub-acute member of this group, is that which, as we just observed, is symptomatised by the deafness and fulness in the ears which many people complain of when suffering from an acute cold in the head. The succulent tissues in the walls of the Eustachian

Normal Membrana Tympani (left), after Politzer.

Retracted Membrane (left) Politzer, by Dalty.

tube are swollen, and its calibre is more or less obstructed by mucus, ventilation and drainage of the tympanic cavity being at the same time temporarily arrested. As a rule this tumefaction and occlusion pass off, as the nasal catarrh or rhinitis subsides, the hearing power not being permanently interfered with; but the more chronic the head cold is allowed to become, or the more recurrent the attacks of a similar kind, the greater is the risk of some secondary changes taking place.

The deafness in this instance is due, partly to the contained secretions in the tympanum, which may vary greatly in amount, and partly to an interference with the vibrating property of the membrana tympani consequent upon its "retraction."

**Indrawing of the Tympanic Membrane.**—This condition, that obtains so long as there is a preponderating balance of atmospheric pressure on the outer side of the membrane, is one of which it will be convenient here to speak. Some degree of indrawing, together with dry cupping of the mucous linings, must result from the exhaustion and "rarefaction" of the air in the nasopharynx and middle ear, on each act of inspiration, when the nasal passages are stenosed by "cold," or other causes. But more direct pressure is brought to bear upon the membrane when the tube itself is blocked, as the air confined in the tympanic cavity soon becomes absorbed by the blood vessels. In this manner a partial vacuum is created, unless an effusion of fluid from the turgid vessels and capillaries takes the place of the air that has disappeared. Eventually, retraction of the drumhead may be rendered permanent by the action of the shortened tensor tympani muscle. Continued pressure externally, has the effect in some subjects of causing the membrane to become thinned, or atrophied, in spots, or localised patches, which look like cicatrices.

## Figures and Tables

**Figure f1:**
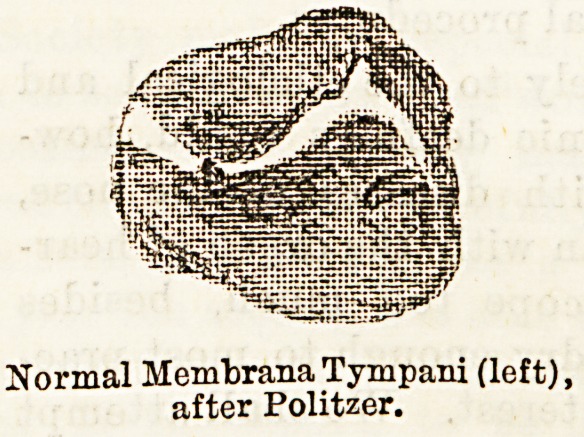


**Figure f2:**